# Grapefruit By-Products as a Sustainable Source of Bioaccessible Polyphenols with In Vitro Neuroprotective Potential

**DOI:** 10.3390/ijms27073140

**Published:** 2026-03-30

**Authors:** Laura Soriano-Romaní, Elisa Gallego, Marc Segarra-Mondéjar, Noelia Teruel, Alejandra Hernández-Bueno, Alessandro Colletti, María Celeste Ruiz-Aracil

**Affiliations:** 1 AINIA, C/Benjamín Franklin 5-11, 46980 Valencia, Spain; 2 Marenostrumtech S.L., C/del Comercio 5, 03380 Alicante, Spain; 3 Department of Science and Drug Technology, University of Turin, 10125 Turin, Italy

**Keywords:** citrus paradisi, agro-industrial residues, antioxidant activity, oxidative stress, simulated digestion, neuronal cells

## Abstract

Cognitive decline and neurodegenerative disorders represent a growing global health challenge, while effective preventive strategies remain limited. Citrus by-products, particularly grapefruit residues, constitute a rich and underexploited source of polyphenols, including flavonoids with reported antioxidant and neuroprotective properties. In this study, grapefruit-derived stabilized extract (GDSE) was evaluated using an in vitro neuronal model combined with dynamic simulated gastrointestinal digestion to assess the bioaccessibility and retained biological activity of key polyphenolic compounds. The soluble intestinal fraction of the digested formulation significantly reduced oxidative stress in dopaminergic-differentiated SH-SY5Y cells and was associated with changes in the expression of genes associated with neurotrophic support, dopaminergic signalling, and neuronal survival. In parallel, simulated digestion preserved a substantial proportion of major flavonoids, such as naringin and narirutin. Consequently, GDSE retained a moderate level of bioaccessible polyphenols and flavonoids, supporting the maintenance of its biological activity after digestion. Overall, these findings indicate that the formulation retains measurable bioactivity after simulated gastrointestinal digestion and modulates molecular markers associated with neuronal survival in vitro. While further in vivo and clinical studies are needed to fully establish its relevance for neuroprotection, the findings provide evidence that grapefruit-derived polyphenolic preparations could represent a potential source of bioactive compounds for further investigation as nutraceutical ingredients.

## 1. Introduction

Cognitive decline and neurodegenerative disorders are becoming increasingly prevalent health challenges, while effective preventive strategies remain limited [1]. Dietary bioactive compounds, particularly polyphenols, are gaining attention for their capacity to modulate oxidative stress, neuroinflammation and mitochondrial function, thereby supporting neuronal function. Among dietary sources, citrus fruits are especially rich in flavonoids and other phenolic compounds, which contribute to their antioxidant and neuroprotective properties [2,3].

In addition to their antioxidant properties, flavonoids exert biological effects through the modulation of intracellular signaling pathways and gene expression. Beyond direct scavenging of reactive oxygen species (ROS), they regulate redox homeostasis by activating endogenous antioxidant responses, particularly via the Nrf2 pathway. Flavonoids also influence key signaling cascades involved in cellular stress and survival, including PI3K/Akt, MAPK, and NF-κB pathways, thereby modulating inflammation, apoptosis, and mitochondrial function. These coordinated molecular actions provide a mechanistic basis for their reported neuroprotective potential across experimental models [4].

Key citrus flavonoids have demonstrated strong neuroprotective potential in preclinical studies. Nobiletin, a polymethoxylated flavone, has been shown to counteract amyloid-β-induced neuronal dysfunction in SH-SY5Y cells and to improve memory and neurogenesis in mouse and rat models of Alzheimer’s disease [5,6,7,8]. Similarly, the flavanone glycosides naringin and narirutin have been reported to protect primary neuronal cultures against oxidative stress and excitotoxic insults in vitro, while enhancing neuronal survival in different rodent models of ageing and neurodegeneration [9,10,11,12,13]. Another polyphenolic compound, gallic acid, exhibits broad neuroprotective effects, including the reduction of reactive oxygen species in vivo [14,15,16].

In addition, growing evidence suggests that combinations of structurally related polyphenols may exert synergistic effects, enhancing their biological activity beyond that of individual compounds. These interactions may involve the coordinated modulation of multiple cellular pathways, including oxidative stress responses, inflammation, and intracellular signalling networks [4]. Collectively, these mechanisms provide a framework for understanding the biological activity of polyphenols and support their potential application in nutraceutical strategies targeting cognitive health.

Evidence from human studies further reinforces the cognitive benefits associated with flavonoids and other polyphenolic compounds [17]. Although large-scale clinical trials remain limited, epidemiological and clinical data suggest that regular consumption of citrus fruits, juices, or extracts is associated with improvements in memory, learning, and executive function, particularly in middle-aged and older adults [18,19,20,21,22,23]. Together, these findings highlight citrus flavonoids as promising dietary components for supporting healthy cognitive ageing and mitigating neurodegenerative decline.

Beyond their biological activity, citrus-derived polyphenolic compounds also represent an opportunity for the sustainable valorisation of agro-industrial residues. Citrus processing generates substantial amounts of residual plant materials, particularly peels, which are enriched in flavonoids and other phenolic constituents. The recovery and reuse of these processing residues can reduce environmental burden while enabling the development of value-added ingredients for functional foods and dietary supplements [4,24]. Previous studies have highlighted the potential of citrus processing residues as sources of antioxidant compounds with neuroprotective properties [24,25]. Grapefruit, in particular, generates large quantities of residual materials during industrial processing, including peels and pulp fractions rich in flavonoids, phenolic acids, and essential oils, which can be repurposed into ingredients with antioxidant, anti-inflammatory, and antimicrobial activities [26,27]. In this context, the present work focuses on the valorisation of grapefruit processing residues through bioactive upcycling and in vitro bioactivity assessment.

Despite the growing body of literature on citrus polyphenols, limited information is available regarding the bioaccessibility and retained neuroactive potential of grapefruit-derived compounds following gastrointestinal digestion, particularly when derived from sustainably sourced processing residues.

Accordingly, the present study investigates the neuroactivity of grapefruit-derived polyphenolic preparations, including a newly developed stabilised formulation, using in vitro neuronal models combined with simulated gastrointestinal digestion. Neuroprotection-associated molecular responses were evaluated in dopaminergic-differentiated neuronal cells following dynamic in vitro digestion, enabling the identification of formulations that retain bioactivity after this physiological process.

## 2. Results

### 2.1. Antioxidant Capacity of Grapefruit Extract in Neuronal Cells

Grapefruit extract was chosen as the principal focus of this study due to its distinctive composition, notably its abundance of flavanones, such as naringin and narirutin, and the methoxyflavone nobiletin, all of which are recognized for their antioxidant and anti-inflammatory properties [10,28,29,30]. Prior to assessing their specific bioactivity, it was necessary to ensure that grapefruit extracts were not toxic for the cellular models employed.

**Figure 1:**
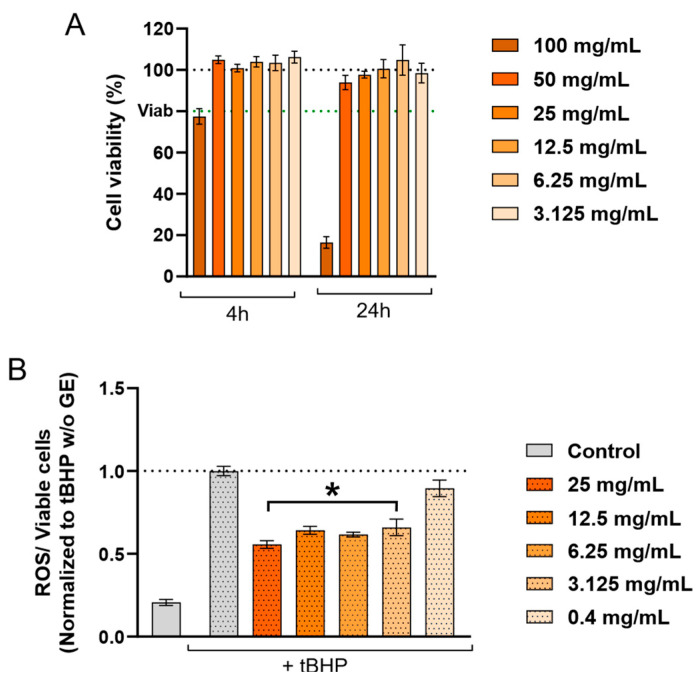
Biocompatibility and antioxidant activity of grapefruit extract (GE) in SH-SY5Y cells. (**A**) Cell viability assessed after exposure to the indicated concentrations of grapefruit extract for 4 or 24 h. The black dotted line represents 100% cell viability, while the green line represents 80% (*n* ≥ 3). (**B**) Intracellular ROS levels in SH-SY5Y cells treated with the selected non-cytotoxic extract concentrations for 4 h. Oxidative damage was induced by incubation with tBHP 100 µM for 2 h. Data are normalized to tBHP-treated cells without extract, set to 1 (*n* ≥ 5). Results are presented as mean ± SEM of at least three independent experiments. One-way ANOVA followed by Tukey’s post hoc test. Significance is indicated as: * *p* ≤ 0.05.

To establish suitable experimental conditions, cytotoxicity assays were first conducted to determine extract concentrations that preserved cell viability and avoided confounding effects related to cytotoxicity (Figure 1A). Non-toxic concentrations were subsequently applied to the evaluation of antioxidant capacity in the human neuroblastoma SH-SY5Y cell line, a neuronal model commonly used to study neuronal function and responses to oxidative stress [31,32]. As shown in Figure 1B, cells exposed to concentrations of 3.125 mg mL^−1^ or higher exhibited lower ROS levels following oxidative damage. These findings suggest a potential capacity of grapefruit extracts to modulate oxidative stress in neuronal cells, though further studies are required to fully characterise their effects and underlying mechanisms.

### 2.2. Bioaccessibility of Grapefruit Extract-Based Formulation

Based on the bioactivity results obtained with grapefruit extracts, the company Marenostrumtech S.L. (Alicante, Spain) developed a new nutraceutical formulation, GDSE. This powder-based formulation offers several practical advantages over liquid extracts, including improved preservation and simplified storage and handling, which may facilitate potential consumption and commercial use.

A critical aspect in the development of functional ingredients is ensuring the bioaccessibility of the active compounds. Since GDSE is intended for oral administration, it is crucial to assess whether it retains the same functional properties as the original extracts after digestion. To this end, GDSE was subjected to simulated gastrointestinal digestion using a dynamic digestion system. This approach allows evaluation of the bioactive potential of the formulations under conditions that approximate physiological use. Following simulated gastrointestinal digestion, the main bioactive constituents of GDSE showed moderate preservation. While flavanones naringin and narirutin showed minimal losses, the methoxylated flavone nobiletin and gallic acid, a polyphenolic acid, exhibited a moderate reduction in bioaccessibility (Table 1). Collectively, these results indicate that the GDSE formulation may maintain the availability of key flavonoids and polyphenols following simulated gastrointestinal digestion.

### 2.3. Antioxidant Capacity of Grapefruit Extract-Based Formulation in Neuronal Cells

**Figure 2:**
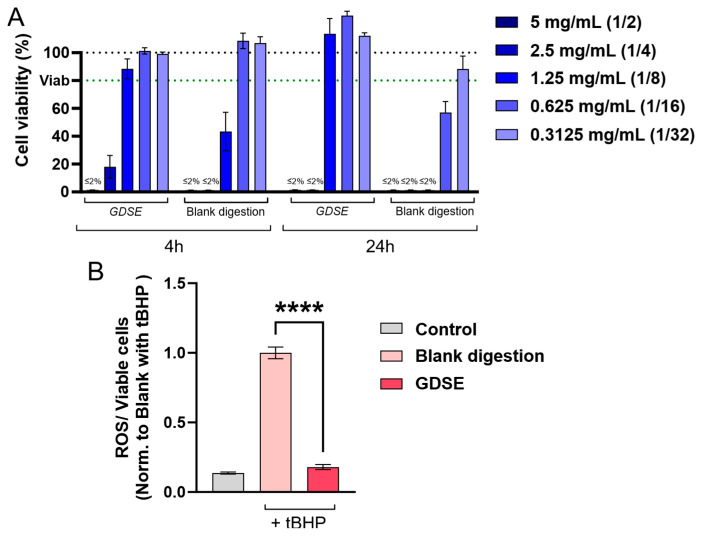
Biocompatibility and antioxidant activity of digested extract-based formulation in SH-SY5Y cells. (**A**) Cell viability assessed after exposure to the indicated dilutions of GDSE and blank digestions for 4 and 24 h. The black dotted line represents 100% cell viability, while the green line represents 80% (*n* ≥ 6). (**B**) Intracellular ROS levels in SH-SY5Y cells treated with the selected non-cytotoxic dilution (1/16, corresponding to a concentration of 0.625 mg mL^−1^) of sample digestions for 4 h. Oxidative damage was induced incubating with tBHP 100 µM for 2 h. Data are normalized to tBHP-treated cells incubated with blank digestion, set to 1 (*n* ≥ 5). Results are presented as mean ± SEM of at least three independent experiments. Two-tailed Student’s *t*-test. Significance is indicated as: **** *p* ≤ 0.0001.

As in previous experiments, cytotoxicity assays were first performed to identify the proper dilution of the digested solution of the digested formulation that maintained cell viability (Figure 2A). A non-toxic 1/16 dilution was selected for subsequent assessment of antioxidant capacity in SH-SY5Y cells. Exposure to the digested preparation resulted in reduced ROS levels following tBHP-induced oxidative stress (Figure 2B). ROS levels were largely restored to values comparable with the untreated control, suggesting that the main bioactive constituents of the formulation retain their antioxidant potential even after simulated gastrointestinal digestion.

### 2.4. Neuroprotection-Associated Responses Induced by a Grapefruit Extract-Based Formulation

**Figure 3:**
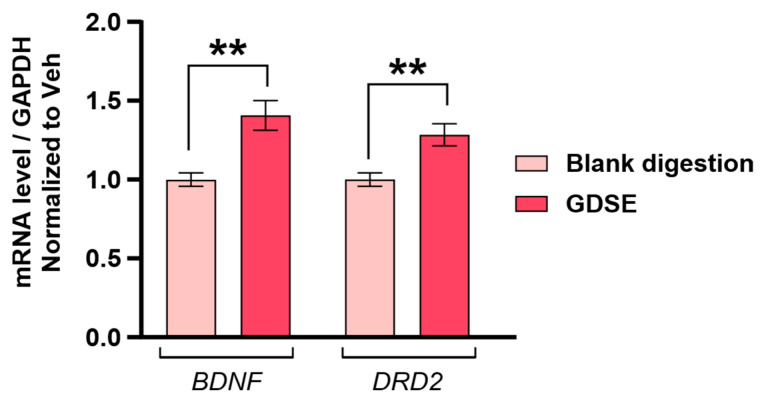
Digested GDSE solution stimulates the synthesis of genes related to neuroprotection in SH-SY5Y cells. mRNA expression levels of *BDNF* and *DRD2* after incubation of SH-SY5Y cells in the presence of a non-cytotoxic dilution (1/16, corresponding to a concentration of 0.625 mg mL^−1^) of the indicated sample digestions during a 4-h period (*n* ≥ 6). Data are presented as mean ± SEM of at least three independent experiments. Two-tailed Student’s *t*-test adjusted for multiple comparisons using the Holm-Šídák method. Significance is indicated as: ** *p* ≤ 0.0201.

Neuronal function depends on the ability of cells to maintain redox homeostasis and to activate survival pathways in response to stress [33]. Key mediators include brain-derived neurotrophic factor (BDNF), which plays a key role in synaptic plasticity, learning, and memory and dopamine receptor D2 (DRD2), essential for dopaminergic neurotransmission. Altered expression of these genes is associated with neurodegeneration and stress-induced neuronal dysfunction [34,35,36]. To investigate whether digested GDSE was able to stimulate molecular responses associated with neuronal survival, *BDNF* and *DRD2* expression was assessed after 4 h of incubation of dopaminergic-differentiated SH-SY5Y cells in the presence of this digested formulation. As shown in Figure 3, both genes were upregulated relative to cells treated with the digestion blank, indicating an early activation of neurotrophic and dopaminergic pathways.

**Figure 4:**
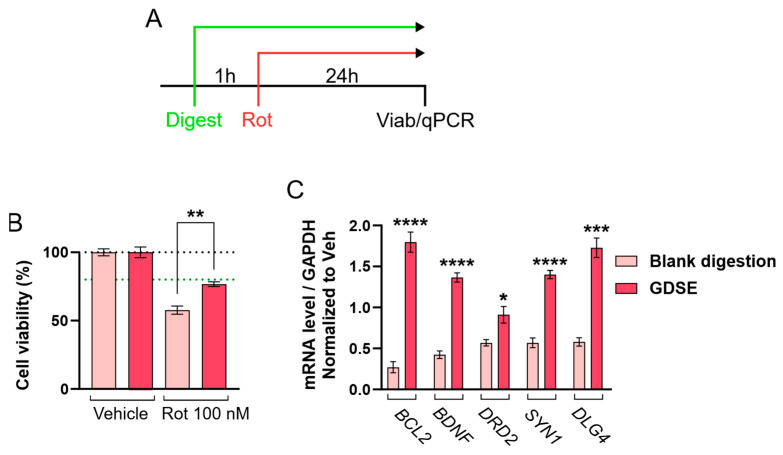
Biocompatibility and neuroprotection-associated responses of digested GDSE solution in SH-SY5Y cells. (**A**) SH-SY5Y cells were pre-incubated for 1 h with non-cytotoxic dilution (1/32, corresponding to a concentration of 0.3125 mg mL^−1^) of the formulation, followed by exposure to rotenone (100 nM) for 24 h (*n* ≥ 6). (**B**) Cell viability of SH-SY5Y cells incubated in the presence of the indicated non-cytotoxic dilution (1/32, corresponding to a concentration of 0.3125 mg mL^−1^) of sample digestions following neuroprotection assay described in (**A**). Each condition is normalized to its viability in the absence of rotenone. (**C**) mRNA expression levels of markers associated with neuronal survival and functional integrity were assessed following the protocol summarized in panel (**A**) (*n* ≥ 4). Data are presented as mean ± SEM of at least three independent experiments. One-way ANOVA followed by Tukey’s post hoc test (**B**) and two-tailed Student’s *t*-test adjusted for multiple comparisons using the Holm-Šídák method (**C**). Significance is indicated as: ** *p* ≤ 0.01 (**B**) or * *p* ≤ 0.0332, *** *p* ≤ 0.0002, **** *p* ≤ 0.0001 (**C**).

Taking this into account, and considering that several flavonoids present in the formulation have previously been reported to exhibit antioxidant and neuroprotective effects [9,10,12,28,37], rotenone, a mitochondrial complex I inhibitor commonly used to model neurodegeneration [38], was employed to induce neuronal damage (Figure 4A). Prior to evaluating neuroprotection-related responses, a 24 h biocompatibility assay was conducted to determine the non-toxic dilution of the digested samples (Figure 2A). Subsequently, cell cultures were pre-incubated for one hour with 1/32 non-toxic dilution before being exposed to a low concentration of rotenone (100 nM) for 24 h. Following incubation with rotenone and the digested GDSE, cell viability was assessed. The selected rotenone concentration (100 nM) and exposure duration (24 h) were based on previous studies reporting reproducible mitochondrial stress and moderate neuronal damage without extensive cytotoxicity. Pre-treatment with the digested product resulted in a mean increase of 16.77% in cell viability compared with rotenone alone (Figure 4B), suggesting that the digested preparation is associated with increased neuronal viability under these experimental conditions.

To explore potential mechanisms, the expression of genes involved in neuronal survival, synaptic integrity, and neurotransmission (*BCL2*, *BDNF*, *DRD2*, *SYN1*, and *DLG4*) was analysed in cells exposed to rotenone (Figure 4C). *BCL2* (B-cell lymphoma 2), *SYN1* (Synapsin I), and *DLG4* (Discs large homolog 4) were included as representative markers of anti-apoptotic signalling and synaptic function. Cells treated with digested GDSE showed higher expression levels of these genes compared with the digestion blank, with most transcripts exceeding levels observed in non-damaged controls. This pattern suggests a coordinated molecular response associated with neuronal survival involving anti-apoptotic (*BCL2*), neurotrophic (*BDNF*), and synaptic maintenance (*DRD2*, *SYN1*, *DLG4*) pathways.

Altogether, these results suggest that, in this cellular model, bioaccessible constituents from the digested formulation are associated with molecular responses related to neuronal survival and synaptic integrity.

## 3. Discussion

The present study provides evidence that GDSE retains measurable bioactivity associated with neuroactive pathways in vitro following dynamic simulated gastrointestinal digestion. The use of a stabilized format allowed the evaluation of bioaccessible polyphenolic fractions under conditions that partially approximate oral consumption, addressing a key limitation of many in vitro studies relying solely on non-digested extracts. The stabilized formulation was primarily designed to preserve compound integrity and handling properties rather than to modulate release kinetics, which were not directly assessed in this study.

At the molecular level, short-term exposure (4 h) of SH-SY5Y neuronal cells to GDSE after digestion resulted in upregulation of *BDNF* and *DRD2* expression, suggesting that bioaccessible components can activate neurotrophic and dopaminergic signalling pathways. Antioxidant effects were also observed at this early time-point, with a reduction in reactive oxygen species (ROS), supporting the idea that the bioaccessible flavonoids and polyphenols can mitigate oxidative stress in neuronal cells in vitro. Observed reductions in specific compounds after simulated digestion may reflect a combination of chemical degradation, interactions with the digestion matrix, and incomplete solubilization, rather than irreversible loss of the polyphenolic compounds.

Upon exposure to rotenone, cells pre-treated with GDSE exhibited increased viability and enhanced expression of genes associated with neuronal survival (*BCL2*), neurotrophic support (*BDNF*), and synaptic maintenance (*DRD2*, *SYN1* and *DLG4*). Transcript-level changes observed in this study should be interpreted as early molecular indicators of cellular responses rather than direct evidence of functional or phenotypic neuronal outcomes. These findings suggest that the bioaccessible fraction modulates coordinated molecular responses associated with neuronal survival under stress conditions. These observations should not be interpreted as direct evidence of functional neuroprotection, but rather as indicative of early cellular responses under the experimental conditions employed.

It should be noted that polyphenolic compounds may directly interfere with fluorogenic probes such as DCFDA (2′,7′-dichlorodihydrofluorescein diacetate). Although cell-free controls were included to account for non-specific signal generation, confirmation using complementary oxidative stress markers (e.g., glutathione levels or antioxidant enzyme activity) would further strengthen these observations.

From a mechanistic perspective, these findings align with previous in vivo evidence that citrus flavonoids such as naringin, narirutin, and nobiletin can regulate neurotrophic pathways, including *BDNF* expression, attenuate oxidative and endoplasmic reticulum stress, and support neurogenesis in rodent models of neurodegeneration and brain aging [5,6,7,8,9,10,11,37]. Collectively, these observations provide mechanistic support for neuroprotection-associated molecular responses in neuronal cell models, suggesting that bioaccessible flavonoids contribute to neuronal survival, at least in part through modulation of neurotrophic and antioxidant pathways [6,9,10,11,12,30,39,40,41,42]. These observations support the potential of flavonoid-containing preparations for further investigation as dietary strategies to promote neuronal survival.

Beyond its biological efficacy, the development of this preparation supports the sustainable valorisation of grapefruit processing residues. By valorising grapefruit processing residues, typically discarded, the approach reduces waste and supports sustainable production. Grapefruit by-products represent a substantial proportion of the fruit and are rich in bioactive compounds, including polyphenols, flavonoids, and essential oils [24,27].

While these findings provide initial insights into the neuroprotective potential of bioaccessible citrus flavonoids, several limitations of the present study must be considered. First, it is important to distinguish between bioaccessibility and in vivo bioavailability. In this study, bioaccessibility refers to the fraction of compounds potentially available for intestinal absorption after digestion, but it does not account for further physiological processes. In addition, the concentrations used exceed those expected under physiological conditions and were selected within an exploratory framework. Therefore, the observed effects should be interpreted as indicative of biological activity under controlled in vitro conditions rather than direct evidence of in vivo efficacy. Second, the study was conducted exclusively in a single neuronal cell line (SH-SY5Y), which, despite being widely used in neurodegeneration research [31,32], does not fully recapitulate the synaptic, glial, or vascular complexity of the human brain. Third, functional neuronal outcomes, such as calcium signalling, mitochondrial function, or neuronal morphology, were not assessed, limiting the interpretation of the observed molecular changes. Fourth, the study did not include a direct evaluation of absorption, metabolic processing, or hepatic transformation of the bioactive compounds. Although the ability of these compounds to cross the blood–brain barrier was not evaluated in the present study, previous in vitro and in vivo studies have demonstrated that certain flavonoids can penetrate this barrier [43,44,45].

Accordingly, further validation using additional cellular models, functional assays, and in vivo systems will be required to confirm the translational relevance of these observations, and the present study should be considered strictly exploratory in nature.

In summary, this work highlights the potential of a grapefruit-derived powdered formulation to deliver bioaccessible flavonoids and polyphenols with antioxidant and neuroactivity-associated properties in neuronal cell models, while also illustrating a sustainable approach to the valorisation of fruit by-products. These findings should be interpreted strictly within an exploratory in vitro framework and are intended to inform mechanistic understanding rather than to predict in vivo efficacy or functional outcomes.

## 4. Methods and Materials

### 4.1. Sample Preparation for In Vitro Screening

GDSE, a stabilized powdered formulation commercially available as Methoxymemory®, was provided by Marenostrumtech S.L. (Alicante, Spain). It was developed to improve the handling, standardization, and technological applicability of grapefruit-derived polyphenolic extracts. While the specific formulation process is proprietary, the technological rationale is based on protecting sensitive flavonoids from oxidation and degradation, improving solubility and enabling dose standardization and industrial scalability. Compared with liquid extracts, this approach facilitates longer shelf-life, easier storage and transport, and greater reproducibility in functional applications. The formulation was therefore selected as a representative stabilized format for evaluating the retention of bioactivity after gastrointestinal digestion.

GDSE was used as received in all experiments and, prior to exposure to cell models, extracts were filtered through a 0.45 µm filter. They were then diluted in cell culture medium, initially at 200 mg mL^−1^, and solubilised at 37 °C with gentle agitation. Solutions were inspected visually to ensure the absence of turbidity or precipitation, and pH was measured for each dilution. Initial screening assays were conducted at 100 mg mL^−1^, based on the crude liquid extract, while accounting for minor differences in total solids among the samples. Cell treatments using digested samples were performed based on fixed dilution factors of the soluble intestinal fraction rather than normalization to individual compound concentrations, allowing comparative evaluation of retained bioactivity under standardized exposure conditions. This preparation ensured consistent and reproducible conditions for subsequent in vitro evaluation of bioactivity.

The concentrations employed in the in vitro assays exceed those expected to be achieved in human plasma following dietary intake and were selected within an exploratory screening framework. Such concentrations are commonly used in cell-based models to identify biological responsiveness and potential mechanisms of action, rather than to predict in vivo efficacy.

### 4.2. Cell Lines and Culture Conditions

The human neuroblastoma cell line SH-SY5Y was obtained from the European Collection of Authenticated Cell Cultures (ECACC, Salisbury, UK). Cells were maintained in a 1:1 mixture of Ham’s F12 (ECACC, Salisbury, UK) and Eagle’s Minimum Essential Medium (EMEM; ATCC, Manassas, VA, USA), supplemented with 2 mM L-glutamine (HyClone, Thermo Fisher Scientific, Waltham, MA, USA), 1% non-essential amino acids (NEAA; HyClone, Thermo Fisher Scientific, Waltham, MA, USA), 15% fetal bovine serum (FBS; Biowest, Nuaillé, France), and 1% penicillin/streptomycin (PAN-Biotech, Aidenbach, Germany). For neuronal differentiation, cells were treated with 10 µM all-trans retinoic acid (RA; Sigma-Aldrich, St. Louis, MO, USA) in medium containing 2% FBS for 6 days, with medium replacement every 2–3 days, to induce a cholinergic phenotype. For dopaminergic differentiation, RA-treated cells were subsequently exposed to 100 nM phorbol 12-myristate 13-acetate (PMA; Sigma-Aldrich, St. Louis, MO, USA) in medium containing 0.2% FBS for 2 additional days, with a single medium change. This sequential protocol yields a neuronal-like phenotype with dopaminergic characteristics, as previously reported.

### 4.3. Dynamic Gastrointestinal Digestion Simulator (DigestSim^®^)

DigestSim^®^ (AINIA, Paterna, Valencia), the dynamic gastrointestinal simulator, consists of two jacketed compartments representing the stomach and the small intestine, both maintained at 37 °C via water circulation. pH in both compartments is continuously monitored and automatically adjusted through controlled addition of HCl in the gastric chamber and NaHCO_3_ in the intestinal chamber. The system operates in continuous mode under computer control, enabling automated regulation of digestive secretions and transit flows according to human physiological data, ensuring stable and reproducible experimental conditions. In addition, the dynamic digestion model represents an average adult gastrointestinal profile and does not account for interindividual variability in digestive conditions.

All digestive solutions were prepared on the day of the assay and kept on ice until gradual addition. Oral digestion was simulated using 300 mL of a salivary solution at 37 °C containing α-amylase (from *Aspergillus oryzae*; Sigma-Aldrich, St. Louis, MO, USA), electrolytes (NaCl, CaCl_2_, NaHCO_3_; Scharlab, Barcelona, Spain), and the test extract. The solution was adjusted to pH 6.8, homogenized for 2 min, and degassed with nitrogen for 10 min. This mixture was introduced into the gastric compartment, which contained initial gastric secretions of pepsin (from porcine source; Sigma-Aldrich, St. Louis, MO, USA), lipase (from *Rhizopus* sp.; Amano Enzyme, Nagoya, Japan), and gastric electrolytes. During gastric digestion, gastric secretions (pepsin and electrolytes) were continuously added to the stomach. Gastric pH was dynamically controlled following physiological profiles [46], and peristaltic mixing was performed via paddle agitation. Gastric emptying into the small intestine was gradual, based on a previously established model [47], simulating slow gastric and intestinal transit in adults [46,48].

Small intestinal secretions were introduced via computer-controlled pumps, including a 7% pancreatin solution (from porcine pancreas; Sigma-Aldrich, St. Louis, MO, USA), 4% porcine bile extract (Sigma-Aldrich, St. Louis, MO, USA) for the first 30 min (reduced to 2% thereafter), and an intestinal electrolyte solution. Intestinal pH was maintained at 6.5–7.0 using 1 M NaHCO_3_ (Scharlab, Barcelona, Spain). The accumulated intestinal digest was collected in a chilled container, homogenized at 38–40 °C, and cooled to 25 °C. Temperature adjustments were applied to replicate physiological digestion and to stabilize collected samples prior to downstream analyses.

In this study, bioaccessibility is defined as the fraction of polyphenolic compounds solubilized in the intestinal phase after simulated digestion. The bioaccessible fraction (soluble fraction) was obtained by centrifugation at 4000× *g* for 30 min at 25 °C using a Heraeus Multifuge X3R centrifuge (Thermo Fisher Scientific, Waltham, MA, USA). This fraction represents methoxyflavonoids solubilized in the intestinal lumen and potentially available for absorption. Soluble fractions were collected for all extracts tested, including non-stabilized concentrated citrus extracts and pilot-scale spray-dried extracts stabilized with maltodextrin in duplicate. Each fraction was rapidly frozen at −20 °C until subsequent methoxyflavonoid quantification and bioactivity assessment.

### 4.4. Cell Viability Assay

For biocompatibility, viability was assessed to determine the optimal working concentration of every tested sample. Cells were seeded in 96-well plates and maintained at 37 °C in a humidified atmosphere with 5% CO_2_ for 24 h. Subsequently, increasing concentrations or serial dilutions (1:2 to 1:64) of the test samples or soluble fractions were added and incubated for an additional 24 h. The selection of dilution for each experimental condition was based on cytotoxicity profiles, using the highest non-cytotoxic concentration at each time point. Accordingly, a 1/16 dilution was used for short-term (4 h) exposure, whereas a 1/32 dilution was applied for longer (24 h) incubations to ensure cell viability. For the neuroprotection assays, the same experimental procedure was repeated, inducing neuronal injury by treating the cultures with rotenone (Sigma-Aldrich, St. Louis, MO, USA).

Cell viability was assessed using the AlamarBlue^®^ assay (Life Technologies, Carlsbad, CA, USA) following the manufacturer’s instructions. Fluorescence was measured with a Fluostar Optima spectrofluorometer (BMG Labtech, Ortenberg, Germany) at an excitation wavelength of 540 nm and an emission wavelength of 590 nm. Viability was calculated relative to untreated control cells using Equation (1). Appropriate controls, including cell-free conditions, were included to account for potential background signal and autofluorescence of the tested compounds.

(1) $$Cell\;viability\;\left(\%\right)\;=\;\frac{{Fluorescence\;units}_{\;Sample}}{{Fluorescence\;units}_{\;Control}}\;\times \;100$$

### 4.5. Reactive Oxygen Species (ROS) Assay Kit

As polyphenolic compounds may exhibit intrinsic autofluorescence and interfere with fluorogenic probes such as DCFDA, cell-free controls were included to account for background fluorescence and potential non-specific signal generation. Therefore, ROS measurements should be interpreted as indicative of oxidative status modulation rather than absolute quantification.

ROS levels were assessed using the DCFDA Cellular ROS Assay Kit (Abcam, Cambridge, UK) according to the manufacturer’s instructions, including wells without cells as internal controls. SH-SY5Y cells were seeded in black 96-well plates with a transparent bottom and cultured in high-glucose DMEM (Dulbecco’s Modified Eagle’s Medium) without phenol red (Sigma-Aldrich, St. Louis, MO, USA) supplemented with 10% FBS and 1% penicillin–streptomycin. DCFDA reagent was prepared at a final concentration of 15 µM and added to the cells, which were then incubated for 45 min at 37 °C in the dark. After this period, each well was exposed to its respective sample for 2 h. Oxidative stress was subsequently induced by incubating cells with tert-butyl hydroperoxide (tBHP; Sigma-Aldrich, St. Louis, MO, USA) at a final concentration of 100 µM for an additional 2 h. Fluorescence was measured immediately using a Fluostar Optima spectrofluorometer with excitation and emission wavelengths set at 485 and 520 nm, respectively.

### 4.6. Gene Expression Analysis

The selected genes were chosen to represent complementary aspects of neuronal survival (BCL2), neurotrophic support (BDNF), dopaminergic signalling (DRD2), and synaptic integrity and function (SYN1 and DLG4). Total RNA was extracted from treated cells using the Maxwell automated system (Promega, Madison, WI, USA). Complementary DNA (cDNA) was synthesised from the isolated RNA using the High-Capacity cDNA Reverse Transcription Kit (Applied Biosystems, Waltham, MA, USA). Quantitative reverse transcription polymerase chain reaction (RT-PCR) was then performed on the synthesised cDNA using a 7500 Fast Real-Time PCR System (Applied Biosystems, Waltham, MA, USA). Specific biomarkers were selected for each cell model and amplified using TaqMan Gene Expression Assays (Thermo Fisher Scientific, Waltham, MA, USA), including *GAPDH* (glyceraldehyde-3-phosphate dehydrogenase; Hs02758991_g1), *BDNF* (Hs02718934_s1), *DRD2* (Hs00241436_m1), *BCL2* (Hs00608023_m1), *DLG4* (Hs01555373_m1), and *SYN1* (Hs00199577_m1). *GAPDH* served as the reference housekeeping gene for normalization. Relative gene expression levels were calculated using the 2^−ΔΔCt^ method [49].

### 4.7. Determination of Polyphenols

#### 4.7.1. Determination of Total Phenolic Content (TPC)

The TPC of the extracts was determined using the Folin–Ciocalteu method with slight modifications [50]. Briefly, 0.2 mL of sample was mixed with 0.5 mL of Folin–Ciocalteu reagent (1:1 diluted), 2.5 mL of Na_2_CO_3_ solution (2 N), and 0.8 mL of distilled water. The mixture was incubated for 2 h at room temperature in the dark. Absorbance was measured at 760 nm using a UV–Vis spectrophotometer (U-0080D, Hitachi, Tokyo, Japan). Results were expressed as gallic acid equivalents (GAE) using a gallic acid standard calibration curve (0–10 mg mL^−1^).

#### 4.7.2. HPLC Analysis of Flavonoids

The flavonoid compounds were determined by HPLC as previously reported by Liu et al. [50], with modifications. The analysis was performed using high-performance liquid chromatography with diode array detection (HPLC-DAD) following an in-house validated protocol (MN 0324-1, Marenostrumtech S.L., Alicante, Spain).

Chromatographic analyses were performed using an Agilent 1260 Infinity II HPLC system (Agilent Technologies, Santa Clara, CA, USA) equipped with an autosampler maintained at 4 °C. Separation was carried out on a Tracer Excel 120 ODSA column (Teknokroma, Barcelona, Spain) (250 mm × 4.6 mm, 5 µm particle size) maintained at 40 °C. The injection volume was 10 µL and the flow rate was 0.5 mL min^−1^. The mobile phase consisted of solvent A (water containing 0.05% trifluoroacetic acid (TFA)) and solvent B (acetonitrile). Gradient elution was performed as follows: 0–30 min, 10–25% B; 30–40 min, 25–70% B; 40–50 min, 70–90% B; 50–52 min, 90% B; and 52–54 min, 10% B.

Sample solutions were prepared by dissolving 25 mg of extract in 25 mL of methanol, followed by vortex mixing or ultrasonic treatment to ensure complete dissolution. Quantification was performed using external calibration curves (1–50 ppm) prepared from analytical standards dissolved in methanol. Compound identification was carried out by comparing retention times and UV spectra with those of authentic standards.

Under these chromatographic conditions, narirutin was detected at 283 nm with a retention time of 36.5 min using a narirutin analytical standard (PhytoLab, Vestenbergsgreuth, Germany), naringin was detected at 283 nm with a retention time of 38.4 min using a naringin analytical standard (Sigma-Aldrich, St. Louis, MO, USA), and nobiletin was detected at 330 nm with a retention time of 47.9 min using a nobiletin analytical standard (Sigma-Aldrich, St. Louis, MO, USA). Peak areas were used for quantification using the corresponding calibration curves for each compound.

### 4.8. Statistical Analysis

Data are presented as mean ± standard error of the mean (SEM). All experiments were conducted independently at least twice, with each study including three to four technical replicates per condition. Statistical comparisons between two conditions were conducted using Student’s *t*-test. An F-test was performed to assess the equality of variances; in case variances were significantly different, the Welch correction was applied. When multiple parameters (genes) were tested simultaneously, *p*-values were adjusted for multiple comparisons using the Holm-Šídák method. One-way ANOVA followed by Tukey’s post hoc test was performed in experiments with more than two conditions. Differences were considered statistically significant at *p* ≤ 0.05. The choice of statistical test was based on the number of conditions compared and variance homogeneity. All statistical analyses were carried out using GraphPad Prism version 10.2.3 (GraphPad Software, San Diego, CA, USA).

## 5. Conclusions

This study shows that a powdered grapefruit-derived formulation retains bioactivity associated with antioxidant and neuroprotection-related markers in a neuronal cell model following simulated in vitro gastrointestinal digestion. Its bioaccessible flavonoids and polyphenols support antioxidant activity, neuronal survival, and modulation of neurotrophic and synaptic genes. While these findings are exploratory and require confirmation in additional models in vitro and in vivo, the work also illustrates a sustainable approach to valorising grapefruit by-products, thereby providing a mechanistic basis for further investigation of citrus flavonoids in neuroprotection-related pathways.

## Figures and Tables

**Table 1:** Content and bioaccessibility of major grapefruit flavonoids and total phenolic content (TPC) in GDSE before and after simulated gastrointestinal digestion.

Grapefruit Extract	Naringin	Narirutin	Nobiletin	TPC
Before digestion (mg/g)	4.064	0.364	5.360	3.000
After digestion (mg/g)	3.641	0.343	3.960	2.425
Bioaccessibility (%)	89.592	94.231	73.881	80.833

## Data Availability

The original contributions presented in this study are included in the article. Further inquiries can be directed to the corresponding author.

## Abbreviations

The following abbreviations are used in this manuscript:

ANOVA	Analysis of variance
BCL2	B-cell lymphoma 2
BDNF	Brain-derived neurotrophic factor
cDNA	Complementary DNA
DAD	Diode array detector
DCFDA	2′,7′-dichlorodihydrofluorescein diacetate
DLG4	Discs large homolog 4
DMEM	Dulbecco’s Modified Eagle’s Medium
DRD2	Dopamine receptor D2
ECACC	European Collection of Authenticated Cell Cultures
EMEM	Eagle’s Minimum Essential Medium
FBS	Fetal bovine serum
GE	Grapefruit extract
GAE	Gallic acid equivalents
GAPDH	Glyceraldehyde-3-phosphate dehydrogenase
GDSE	Grapefruit-derived stabilized extract
HPLC	High-performance liquid chromatography
NEAA	Non-essential amino acids
PMA	Phorbol 12-myristate 13-acetate
RA	Retinoic acid
ROS	Reactive oxygen species
RT-PCR	Reverse transcription polymerase chain reaction
SEM	Standard error of the mean
SYN1	Synapsin I
tBHP	tert-butyl hydroperoxide
TFA	Trifluoroacetic acid
TPC	Total phenolic content
UV	Ultraviolet
